# Cerebrovascular Accidents Associated with Sorafenib in Hepatocellular Carcinoma

**DOI:** 10.1155/2011/616080

**Published:** 2011-05-30

**Authors:** Muhammad W. Saif, Iris Isufi, Jennifer Peccerillo, Kostas N. Syrigos

**Affiliations:** ^1^Division of Hematology, Oncology, Department of Medicine, Columbia University, NY 10027, USA; ^2^Section of Medical Oncology, Yale University School of Medicine, New Haven, CT, USA; ^3^Oncology Unit, 3rd Department of Medicine, Athens University School of Medicine, Building Z, Sotiria General Hospital, Mesogion 152, 115 27 Athens, Greece

## Abstract

Sorafenib is an oral angiogenetic multikinase inhibitor approved in the treatment of renal and hepatocellular carcinoma. Bleeding and venous thrombotic events have been described with angiogenetic agents but cerebrovascular accidents are rarely reported. We report two cases of patients with hepatocellular carcinoma who developed a cerebrovascular accident while on sorafenib. Neither patient had any risk factors for the cerebrovascular events apart from gender and age in the second patient. Laboratory data were noncontributory. The head CT scan did not reveal acute abnormalities. No hemodynamically significant stenosis was visible in the carotid ultrasound, and the echocardiogram showed normal size of the heart chambers and normal systolic function of the left ventricle. Sorafenib was discontinued in both cases. Physicians should monitor patients receiving sorafenib for neurologic symptoms, and in the absence of other etiology, prompt discontinuation of this drug should be considered.

## 1. Background

Sorafenib (trade name Nexavar, Bayer Pharmaceuticals, Wayne, NJ, USA) is an orally active multikinase inhibitor that has been approved in the treatment of advanced hepatocellular (HCC) and renal cell carcinoma [1]. Its antineoplastic effect is based on two main mechanisms: the inhibition of both cell proliferation (by inhibiting Raf kinase) and angiogenesis (by inhibiting VEGFR2, VEGFR3, and PDGFR-*β* receptors) [2]. The approval of sorafenib in 2007 for the treatment of HCC was based on the SHARP (Sorafenib in Hepatocarcinoma Carcinoma Assessment Randomized Protocol) study, a multicenter, phase III, double-blind, placebo-controlled trial [3]. The most common adverse reactions observed in patients receiving sorafenib included constitutional symptoms, such as asthenia and fatigue, dermatologic disorders such as hand-foot skin reaction, rash and alopecia, as well as gastrointestinal and liver dysfunction. Hypertension of all grades was reported in 5% and 2% of patients in the sorafenib and placebo group, respectively, (*P* = .05). The difference in the incidence of grade 3 hypertension was not statistically significant (2% versus <1%, *P* = .28) and no grade 4 hypertension reported. The incidences of cardiac ischemia or infarction were similar (3% and 1%) [3]. When data from multiple clinical trials on sorafenib were collected, hypertension crisis, cerebral hemorrhage, myocardial infarction, and chronic heart failure were relatively uncommon (<1%) [4]. However, the documented association between angiogenic inhibitors, such as bevacizumab [5] and sorafenib [6], and thromboembolic and hemorrhagic events, dictates careful consideration during sorafenib administration since the risk in such patients is unknown.

We report two cases of patients with HCC who had a cerebrovascular accident (CVA) while on sorafenib. We studied their medical records to assess risk factors for CVA, including age, sex, smoking, prior CVA, hypertension, diabetes mellitus, hypercholesterolemia, coronary artery disease, peripheral vascular disease, and atrial fibrillation. The chronological relation between sorafenib administration and the CVA was also evaluated.

## 2. Case Presentation

The first patient is a 66 year-old Caucasian male who was diagnosed with well-differentiated HCC in a background of cirrhosis in April 2009. Serology was found positive for hepatitis C, but unaware of the infection. He was had an otherwise unremarkable medical history, he was an ex-smoker (55 pack-years), and was receiving no medication. Based on the presence of vascular invasion and his impaired liver function, the disease was classified as stage C HCC according to the Barcelona Clinic Liver Cancer (BCLC) staging system. He was started on sorafenib at a dose of 400 mg twice daily. Within a month of starting sorafenib, he presented with left facial droop, slurred speech, and left upper extremity weakness. His platelet count was 63,000/mm^3^ and the rest of the laboratory tests were noncontributory.

The second patient is a 75 year-old non-smoker Caucasian male diagnosed with HCC with serology negative for hepatitis B and C. His medical history was unremarkable except for being diagnosed with stage III colon cancer 2 years before and having received adjuvant FOLFOX treatment. The only possible HCC risk factor in the patient's medical history was his exposure to chemical substances while working in plastics industry for many years. Biopsy of the hepatic lesion revealed that it was a second primary malignancy and not a hepatic metastasis from the treated colon cancer. Based on his performance status and the presence of vascular invasion, he was diagnosed with stage C HCC. While on single-agent sorafenib for about 5 weeks at 400 mg twice a day, he presented with confusion, left facial drooping, garbled speech, and urinary incontinence. The platelet count was 366,000/mm^3^.

Neither patient had developed hypertension while on sorafenib nor were they overweight. The head computed tomography (CT) scan was performed with contrast medium in both patients and did not reveal acute abnormalities in either one. No hemodynamically significant stenosis was visible in the carotid ultrasound, and the echocardiogram showed normal size of the heart chambers and normal systolic function of the left ventricle. Sorafenib was discontinued in both cases. Neither patient recovered fully from their neurological symptoms. The acute onset of symptoms as well as the fact that the latter partially improved with no steroid administration support the notion that the neurological presentation was attributed to CVA and not brain metastases. Later on, this was also confirmed in one of the two patients with a brain MRI.

## 3. Discussion

Recent advances in the molecular biology of cancer have led to the introduction of antiangiogenic biologic agents, such as bevacizumab, sunitinib, and sorafenib into clinical trials as well as into clinical setting. These drugs seem to be associated with increased risk of bleeding and arterial and venous thromboembolic events [7]. 

HCC is characterized by the activation of the Raf/MEK/EPK pathway and is a highly angiogenic malignancy. It has been found that multiple factors, such as EGFR, VEGF, and hepatitis C virus, are implicated in the activation of this pathway [8]. Apart from the fact that cerebrovascular events are generally more common in cancer patients [9], the association between HCC and risk of CVA has been poorly studied. Metabolic syndrome is a strong risk factor for HCC even in nonfibrotic liver and is related to underlying vascular pathology [10]. However, our two patients were not characterized by central obesity, they were not diabetic and their lipidemic profile was within normal range. Furthermore, ultrasound revealed no significant carotid disease. Therefore, neither patient had additional risk factors for CVA, other than their gender and the age factor in the second patient. 

The mechanism, by which sorafenib may cause CVA, is not clear, but multiple pathways may be implicated. Firstly, the inhibition of VEGF by sorafenib disrupts the regeneration of endothelial cells causing vascular wall defects and, thereby, the possibility of thrombosis is increased [7]. The production of nitric oxide is also reduced by the inhibition of VEGF, whereas platelet activation and degranulation are induced [11]. CVA during sorafenib treatment may also be associated with uncontrolled hypertension [12], but this was not the case in our two patients. Drug-related cardiac arrhythmias (i.e., atrial fibrillation) could be an underlying cause for acute cerebral events, but, so far, arrhythmia has not been reported in the safety profile of sorafenib [6]. Finally, concurrent chemotherapy may increase the risk for CVA [13] but in our cases, the CVA developed while the patients were on sorafenib monotherapy. 

These two cases suggest the need for careful evaluation of a patient's individual risk for CVA before sorafenib is started and for close monitoring thereafter. Physicians must be alert in order to detect hemostatic complications and neurological symptoms, and they should consider discontinuing sorafenib promptly in absence of other risk factors. Further studies are required to better understand the pathophysiologic mechanisms involved and to define a safe prophylactic strategy. In the meantime, treatment with sorafenib should be selected based on a benefit-risk assessment.
